# Prognostic potential of serum mesencephalic astrocyte-derived neurotrophic factor in acute intracerebral hemorrhage: a prospective observational study

**DOI:** 10.1186/s12883-023-03254-y

**Published:** 2023-06-02

**Authors:** Cheng-Liang Zhang, Ling-Li Fang, Chuan-Liu Wang, Ping Li, Ming Yang, Jian-Wei Xu

**Affiliations:** 1 Department of Neurology, The Quzhou Affiliated Hospital of Wenzhou Medical University, Quzhou People’s Hospital, 100 Minjiang Road, Quzhou, Zhejiang Province 324000 People’s Republic of China; 2 Department of Clinical Pharmacy, The Second People’s Hospital of Yuhang District, 80 Anle Road, Hangzhou, Zhejiang Province 311121 People’s Republic of China; 3 Department of Respiratory and Critical Care Medicine, The Quzhou Affiliated Hospital of Wenzhou Medical University, Quzhou People’s Hospital, 100 Minjiang Road, Quzhou, Zhejiang Province 324000 People’s Republic of China; 4 Department of Clinic, The Quzhou Hospital of TCM, Quzhou TCM Hospital at the Junction of Four Provinces Affiliated to Zhejiang Chinese Medical University, 117 Quhua Road, Quzhou, Zhejiang Province 324000 People’s Republic of China

**Keywords:** Mesencephalic astrocyte-derived neurotrophic factor, Intracerebral hemorrhage, Early neurologic deterioration, Prognosis, Severity

## Abstract

**Objective:**

Mesencephalic astrocyte-derived neurotrophic factor (MANF) expressions are dramatically up-regulated in injured brain tissues, thereby conferring neurological protective effects. We intended to determine significance of serum MANF as a prognostic biomarker of intracerebral hemorrhage (ICH).

**Methods:**

In this prospective, observational study done from February 2018 to July 2021, 124 patients with new-onset primary supratentorial ICH were consecutively enrolled. Also, a group of 124 healthy individuals constituted controls. Their serum MANF levels were detected using the Enzyme-Linked Immunosorbent Assay. National Institutes of Health Stroke Scale (NIHSS) and hematoma volume were designated as the two severity indicators. Early neurologic deterioration (END) was referred to as an increase of 4 or greater points in NIHSS scores or death at post-stroke 24 h. Post-stroke 90-day modified Rankin scale (mRS) scores of 3–6 was considered as a poor prognosis. Serum MANF levels were analyzed using multivariate analysis with respect to its association with stroke severity and prognosis.

**Results:**

Patients, in comparison to controls, displayed markedly elevated serum MANF levels (median, 24.7 versus 2.7 ng/ml; *P* < 0.001), and serum MANF levels were independently correlated with NIHSS scores (beta, 3.912; 95% confidence interval (CI), 1.623-6.200; VIF = 2.394; t = 3.385; *P* = 0.002), hematoma volumes (beta, 1.688; 95% CI, 0.764–2.612; VIF = 2.661; t = 3.617; *P* = 0.001) and mRS scores (beta, 0.018; 95% CI, 0.013–0.023; VIF = 1.984; t = 2.047; *P* = 0.043). Serum MANF levels significantly predicted END and poor 90-day prognosis with areas under receiver operating characteristic curve at 0.752 and 0.787 respectively. END and prognostic predictive abilities were similar between serum MANF levels and NIHSS scores plus hematoma volumes (all *P* > 0.05). Combination of serum MANF levels with NIHSS scores and hematoma volumes had significantly higher prognostic capability than each of them (both *P* < 0.05). Serum MANF levels above 52.5 ng/ml and 62.0 ng/ml distinguished development of END and poor prognosis respectively with median-high sensitivity and specificity values. Using multivariate analysis, serum MANF levels > 52.5 ng/ml predicted END with odds ratio (OR) value of 2.713 (95% CI, 1.004–7.330; *P* = 0.042) and > 62.0 ng/ml predicted a poor prognosis with OR value of 3.848 (95% CI, 1.193–12.417; *P* = 0.024). Using restricted cubic spline, there was a linear correlation between serum MANF levels and poor prognosis or END risk (both *P* > 0.05). Nomograms were well established to predict END and a poor 90-day prognosis. Under calibration curve, such combination models were comparatively stable (using Hosmer & Lemeshow test, both *P* > 0.05).

**Conclusion:**

Increased serum MANF levels after ICH, in independent correlation with disease severity, independently distinguished risks of END and 90-day poor prognosis. Therefore, serum MANF may be a potential prognostic biomarker of ICH.

## Introduction

Acute spontaneous intracerebral hemorrhage (ICH) is a frequently encountered cerebrovascular disease characterized by high morbidity and mortality [1]. National Institutes of Health Stroke Scale (NIHSS) and hematoma volume are two of the commonest prognostic predictors of ICH [2, 3]. Early neurologic deterioration (END) is an often-occurring complication in relation to worse stroke outcome [4]. Therefore, whether predicting END or poor prognosis is of rather importance in ICH monitoring and management [5]. Noteworthily, exploration of biochemical markers (such as C-reactive protein, vascular endothelial growth factor, glial fibrillary astrocyte protein, and S100 calcium binding protein B) has emerged as a research topic with respect to prognostic significance in neurologic field [6, 7].

Clearly, endoplasmic reticulum stress is an important mechanism implicated in secondary brain injury after ICH [8]. Mesencephalic astrocyte-derived neurotrophic factor (MANF) belongs to the neurotrophic factor family [9]. Expression of MANF by neurons were dramatically up-regulated under endoplasmic reticulum stress [10]. Also, MANF expressions were significantly increased in brain tissues of animals subjected to acute brain injury, including ICH [11]. A growing body of experimental data have shown that MANF may confer neuroprotective functions via anti-inflammatory property [11–14]. Intriguingly, a recent study has reported that serum MANF levels were substantially increased in rats with acute ischemic stroke or patients, and elevated levels of MANF were highly correlated with stroke severity of patients [15]. Herein, we attempted to determine whether serum MANF may be a potential prognostic biomarker in relation to hemorrhagic severity, END and prognosis of human ICH.

## Materials and methods

### Study design, participant selection and ethics approval

In this prospective observational study, which was implemented at our hospital from February 2018 to July 2021, patients with new-onset primary supratentorial ICH were consecutively enrolled. Primary ICH were free of brain hemorrhage resulting from congenital or acquired coagulation abnormalities, hemorrhagic transformation of cerebral infarction, moyamoya disease, cerebral aneurysm, cerebral arteriovenous malformation or intracranial tumors. Inclusion criteria included (1) hospital admission within 24 h after stroke symptom onset, (2) age of 18 years or greater, and (3) a conservative treatment of ICH. Exclusion criteria were as follows: (1) history of other neurologic diseases, such as stroke, intracranial tumors and severe traumatic brain injury; (2) other specific diseases or conditions, such as pregnancies, malignancies and autoimmune system diseases; (3) severe cardiac, hepatic, pneumonic or renal dysfunction. Alternatively, a group of healthy volunteers constituted controls. The current study was fulfilled in compliance with the tenets of the Declaration of Helsinki and the protocol was approved by the ethics committee at the Quzhou Affiliated Hospital of Wenzhou Medical University (Quzhou People’s Hospital) (opinion number: LW2021-012). Signed informed consent to participation was acquired from participants and their parents/relatives or controls themselves. No minors were involved in our study.

### Data collection, clinical assessment and outcome analysis

We registered some basic information, including age, gender, body mass index, cigarette smoking and alcohol drinking. Some specific chronic diseases, such as hypertension, diabetes mellitus, hyperlipidemia and coronary heart disease, were inquired. Statins, anticoagulants, antiplatelet agents, antihypertensive drugs, hypoglycemic drugs and insulin were in frequent use and therefore were recorded. The non-invasively measured vital signs included systolic arterial blood pressure and diastolic arterial blood pressures. The severity indicators constituted NIHSS and hematoma volume, which was calculated according to the formula 0.5×a×b×c [16]. Supratentorial ICH comprised lobar and deep bleedings. Some hematomas may be extended into subarachnoidal cavity or intraventricular space, which was distinguished via head computerized tomography scan. When there was an increase of ≥ 4 in the NIHSS score or death within 24 h after admission, END was considered [17]. We made 90-day follow-up to ICH patients using modified Rankin scale (mRS) and mRS scores of 3–6 was referred to as a worse outcome [18].

### Blood collection, sample processing and immune analysis

When ICH patients arrived at emergency room, 5 ml of venous blood was drawn via the antecubital vein and then was put into gel-containing biochemistry tube. When healthy controls were enrolled into this study, venous blood was obtained using similar methods as in ICH patients. After centrifugation at 3500 g for 10 min, serum was placed in Eppendorf tubes and subsequently stored at -80 °C for later MANF measurement. Using the Enzyme-Linked Immunosorbent Assay (Boster Biotechnology, Ltd., Wuhan, China), serum MANF levels were in duplicate determined by the same technician, who was inaccessible to investigatory data. Mean value of two measures was utilized for further analysis.

### Statistical analysis

The SPSS 23.0 software (SPSS Inc., Chicago, IL, USA) and R software (version 3.5.1; https://www.r-project.org) were in use for statistical analysis. Categorical variables were reported in the form of counts (percentages). The Shapiro-Wilk normality test was done to ascertain whether continuous variables were normally distributed. Non-normally and normally distributed variables were summarized as medians (percentiles 25th – 75th) and means (standard deviation, SD) respectively. Applications of the Pearson chi-square test, Mann-Whitney U test and independent sample t test were implemented for comparing two-group differences of categorical variables and non-normally and normally distributed variables respectively. Also, we used another non-parametric test (namely, the Kruskal-Wallis test) for multiple-group comparisons of serum MANF levels. Spearman’s correlation coefficient test was performed to achieve bivariate correlative analysis. The binary logistic regression model and multivariate linear regression model were built for multivariate analysis, wherein, those variables of significant difference in univariate analysis were incorporated. Nomograms were formed to establish prognostic models and the corresponding calibration curves were drawn to evaluate model stability. The receiver Operating characteristic (ROC) curve, which can give the best threshold value, was explicitly used for binary classification. Area under ROC (AUC) was estimated to indicate prognostic efficiency. The two-sided significance level was set at a *P* < 0.05.

## Results

### Study population’s selection and characteristics

**Fig. 1:**
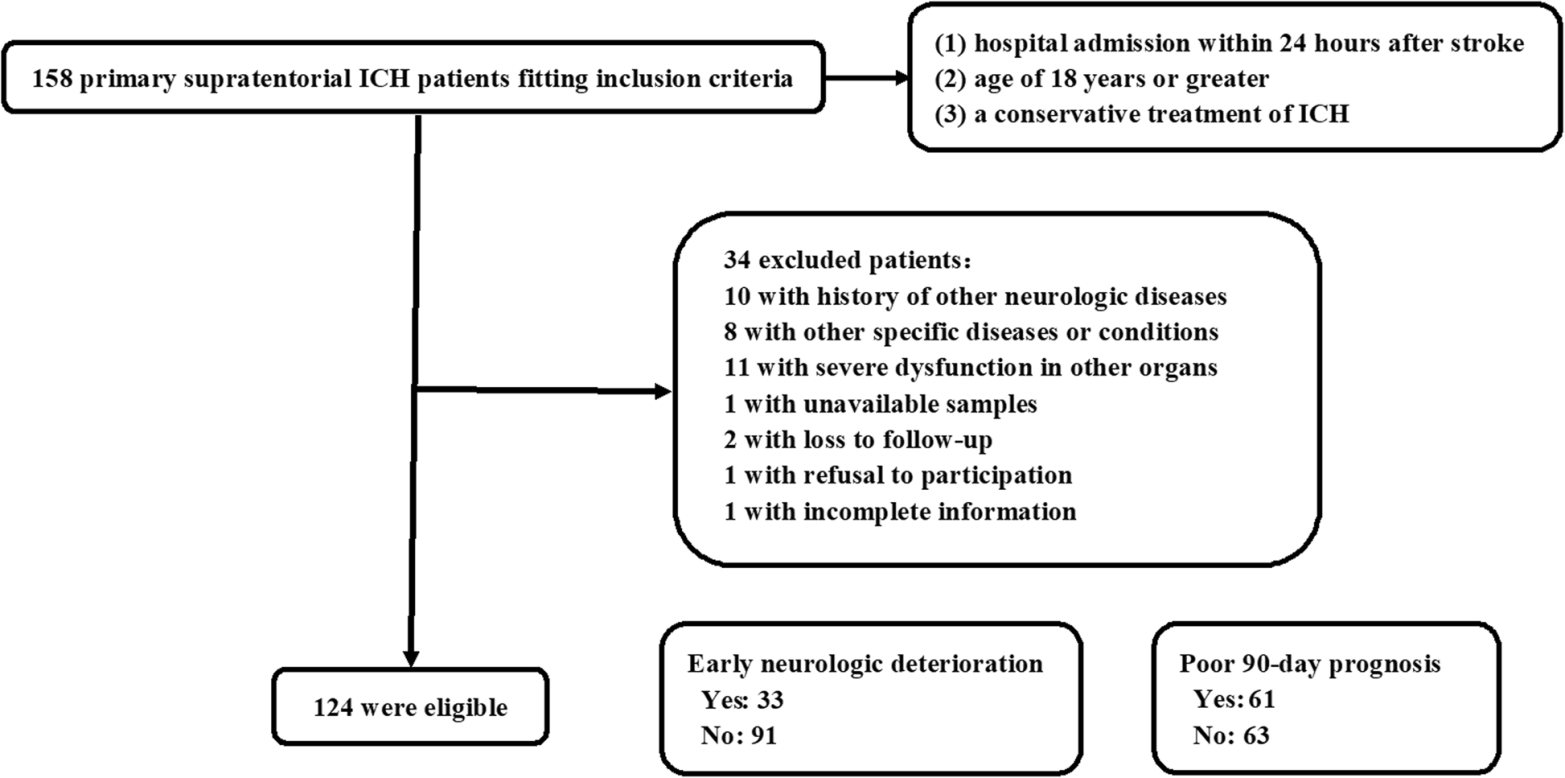
Study workflow to choose qualified patients with acute supratentorial intracerebral hemorrhage. A total of 158 patients with intracerebral hemorrhage got an initial assessment in compliance with inclusion criteria, thereafter there were 34 patients removed from this study because of exclusion criteria and finally 124 patients were analyzed for clinical investigation, among whom, 31 patients suffered from early neurologic deterioration and 61 patients experienced a poor prognosis at 90 days after stroke. ICH indicates intracerebral hemorrhage

In accordance to flowing-diagram outlined in Fig. 1, a total of 124 eligible ICH patients were acquired for final clinical investigation, among whom, there were 33 with END and 61 with poor 90-day prognosis. This cohort of patients, 69 being males and 55 being females. were aged from 38 to 86 years (mean, 61.5 years; SD, 12.2 years). This group of controls, of whom 65 were males and 59 were females, were aged from 29 to 90 years (mean, 60.8 years; SD, 14.1 years). Mean age and gender percentage insignificantly differed between controls and patients (both *P* > 0.05).

Among this cohort of ICH patients, body mass index ranged from 19.1 to 34.4 kg/m^2^, with a mean value of 25.7 kg/m^2^ (SD, 3.4 kg/m^2^). There were 74 hypertensive patients, 28 diabetic patients, 38 hyperlipidemia patients, 9 patients inflicted with coronary heart disease, 44 cigarette smokers and 42 alcohol drinkers. Totally, 21, 5, 16, 67 and 21 patients had medication histories of statins, anticoagulants, antiplatelet agents, antihypertensive drugs and hypoglycemic drugs or insulin respectively. Patients were hospitalized from 0.5 to 24.0 h (median, 9.4 h; percentiles 25th-75th, 5.9–14.3 h) after stroke. Blood samples of patients were obtained from 1.0 to 25.6 h (median, 11.1 h; percentiles 25th-75th, 7.0-15.8 h) after ICH. Systolic arterial pressure and diastolic arterial pressure ranged from 94 to 213 mmHg (mean, 146.4 mmHg; SD, 23.1 mmHg) and from 66 to 116 mmHg (mean, 87.7 mmHg; SD, 10.9 mmHg) respectively. With regard to hematoma location, ratio of lobar to deep hematoma was 0.35 (32/92). Hematomas were extended into intraventricular and subarachnoid space in 32 and 11 patients respectively. With respect to stroke severity, NIHSS scores and hematoma volumes ranged from 0 to 18 (median, 8; lower-upper quartiles, 5–11) and from 3 to 53 ml (median, 16 ml; lower-upper quartiles, 10–25 ml) respectively. As regards laboratory measurements, blood leucocyte count and blood glucose levels ranged from 3.7 to 17.7 × 10^9^/l (median, 8.4 × 10^9^/l; lower-upper quartiles, 6.5–10.5 × 10^9^/l) and from 3.3 to 25.9 mmol/l (median, 11.3 mmol/l; lower-upper quartiles, 9.4–14.6 mmol/l) respectively.

### Serum MANF levels after ICH and its independent correlation with stroke severity

**Fig. 2:**
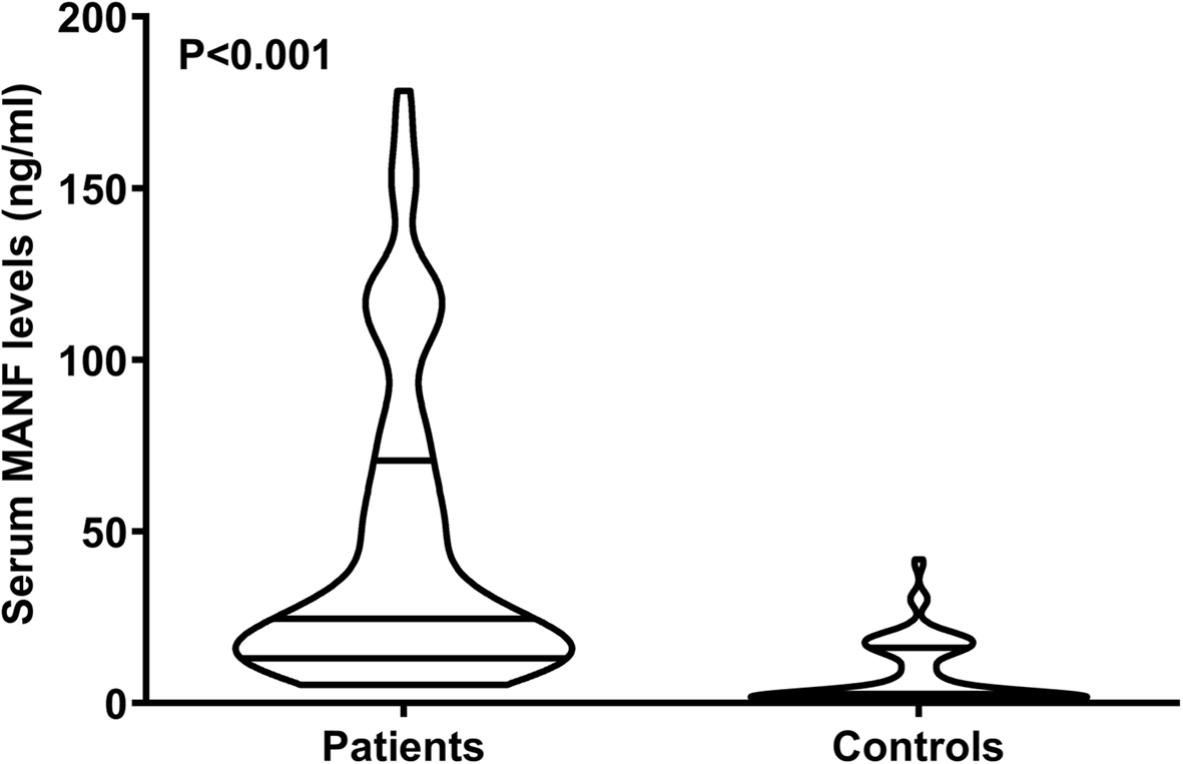
Serum mesencephalic astrocyte-derived neurotrophic factor levels following acute intracerebral hemorrhage. Patients with acute intracerebral hemorrhage, in comparison with healthy controls, had significantly raised serum mesencephalic astrocyte-derived neurotrophic factor levels (*P*<0.001). MANF denotes mesencephalic astrocyte-derived neurotrophic factor

In Fig. 2, patients had dramatically higher serum MANF levels than controls (*P* < 0.001). In order to ascertain relationship between serum MANF levels and other variables in patients with ICH, bivariate correlations were carried out using the Spearman test. Just as listed in Table 1, there was a close correlation between serum MANF levels and extension of hematoma into intraventricular cavity (*P* < 0.05), between serum MANF levels and extension of hematoma into subarachnoidal space, between serum MANF levels and NIHSS scores (*P* < 0.001), between serum MANF levels and hematoma volumes (*P* < 0.001), between serum MANF levels and blood leucocyte levels (*P* < 0.05), as well as between serum MANF levels and blood glucose levels (*P* < 0.05). Using the multivariate linear regression model, wherein the significantly correlative variables were entered, serum MANF levels were independently correlated with NIHSS scores (beta, 3.912; 95% CI, 1.623-6.200; VIF = 2.394; t = 3.385; *P* = 0.002) and hematoma volumes (beta, 1.688; 95% CI, 0.764–2.612; VIF = 2.661; t = 3.617; *P* = 0.001).

**Table 1:** Factors in correlation with serum mesencephalic astrocyte-derived neurotrophic factor levels after acute intracerebral hemorrhage

Variables	*ρ*	*P* value
Age (years)	-0.015	0.870
Gender (male/female)	-0.037	0.687
Body mass index (kg/m^2^)	0.010	0.916
Hypertension	-0.017	0.855
Diabetes mellitus	0.050	0.583
Hyperlipidemia	-0.090	0.321
Coronary heart disease	0.121	0.180
Cigarette smoking	0.092	0.308
Alcohol drinking	0.050	0.579
Pretreatment of statins	-0.098	0.278
Pretreatment of anticoagulation drugs	-0.056	0.540
Pretreatment of antiplatelet drugs	-0.054	0.548
Pretreatment of antihypertensive drugs	0.019	0.832
Pretreatment of hypoglycemic drugs or insulin	0.040	0.657
Admission time (h)	0.049	0.589
Blood-collection time (h)	0.048	0.594
Systolic arterial pressure (mmHg)	-0.072	0.425
Diastolic arterial pressure (mmHg)	-0.022	0.805
Hemorrhage locations (lobar / deep)	0.068	0.450
Extension of hematoma into intraventricular cavity	0.198	0.027
Extension of hematoma into subarachnoidal space	0.196	0.029
NIHSS scores	0.627	< 0.001
Hematoma volume (ml)	0.648	< 0.001
Blood leucocyte count (×10^9^/l)	0.220	0.014
Blood glucose levels (mmol/l)	0.194	0.031

Using Spearman’s correlation coefficient test, correlations were reported as *ρ* values. *NIHSS* indicates National Institutes of Health Stroke Scale

### Serum MANF levels in independent relation to END risk after ICH

A total of 33 ICH patients suffered from END after stroke. Serum MANF levels ranged from 12.7 to 155.0 ng/ml (median, 69.0 ng/ml; upper-lower quartiles, 24.6-118.1 ng/ml) in patients with END risk and from 5.3 to 178.4 ng/ml (median, 21.6 ng/ml; upper-lower quartiles, 12.1–48.5 ng/ml) in those without END risk. Clearly, serum MANF levels were substantially higher in END patients than in those not suffering from END (*P* < 0.001).

**Fig. 3:**
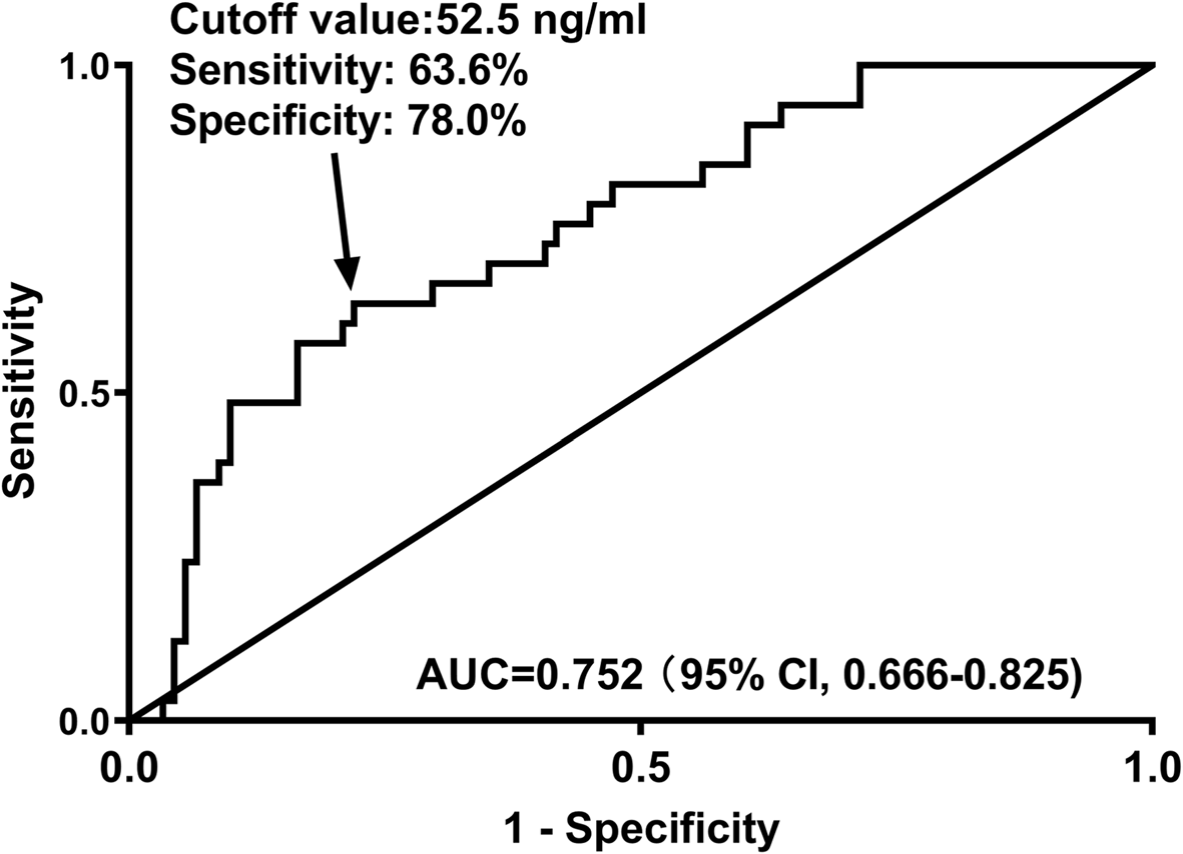
Receiver operating characteristic curve of serum mesencephalic astrocyte-derived neurotrophic factor levels for predicting early neurologic deterioration after acute supratentorial intracerebral hemorrhage. Serum mesencephalic astrocyte-derived neurotrophic factor levels significantly distinguished early neurologic deterioration following acute intracerebral hemorrhage and using Youden method, an optimal value was selected, which predicted such an adverse affair with medium-high sensitivity and specificity values (*P*<0.001). AUC means area under curve; 95% CI, 95% confidence interval

**Fig. 4:**
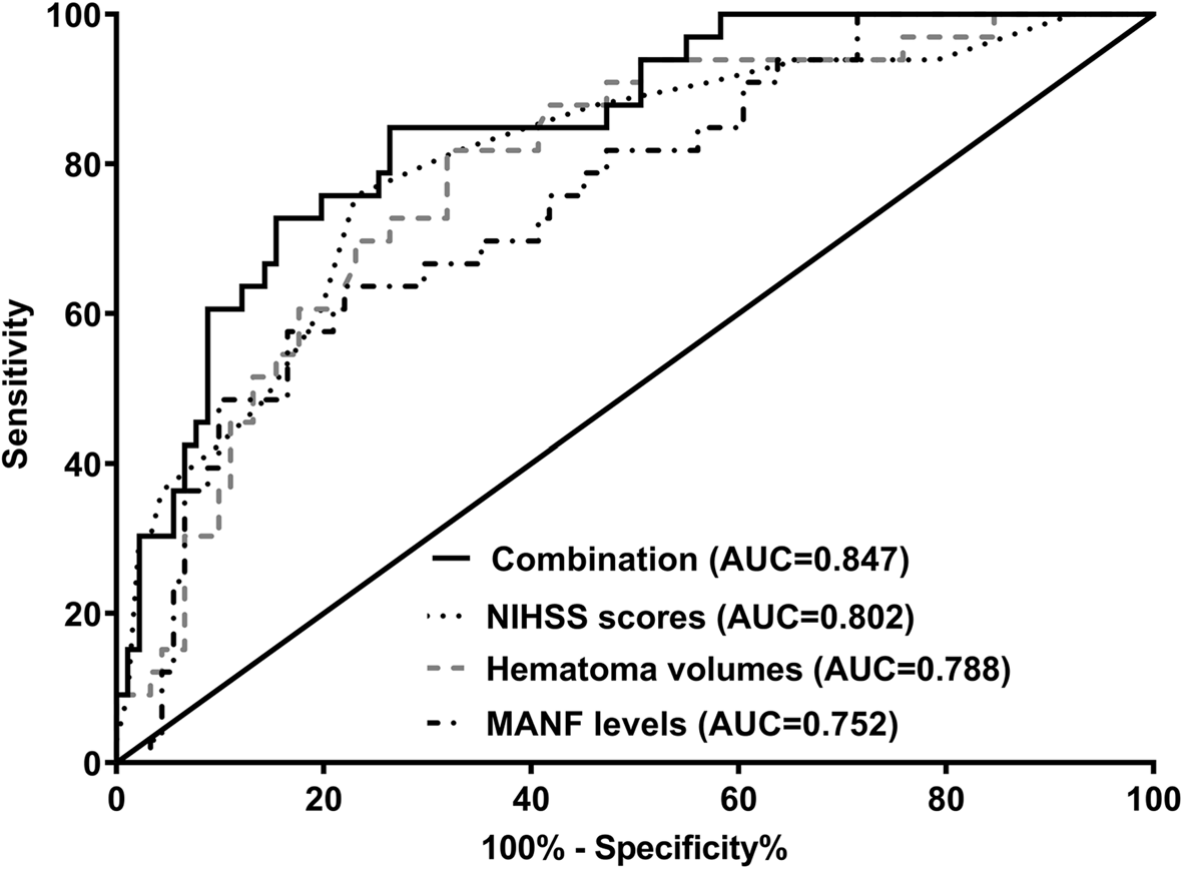
Predictive ability with respect to combination model for early neurologic deterioration after acute supratentorial intracerebral hemorrhage. Predictive ability of serum mesencephalic astrocyte-derived neurotrophic factor levels for early neurologic deterioration resembled those of National Institutes of Health Stroke Scale scores and hematoma volume (both *P*>0.05). Nevertheless, serum mesencephalic astrocyte-derived neurotrophic factor levels combined with National Institutes of Health Stroke Scale scores and hematoma volume showed numerically higher predictive capability than National Institutes of Health Stroke Scale scores and hematoma volume alone (both*P*>0.05). MANF denotes mesencephalic astrocyte-derived neurotrophic factor; NIHSS, National Institutes of Health Stroke Scale; AUC, area under curve

Under ROC curve, Youden method was used to identify serum MANF levels > 52.5 ng/ml distinguished END risk with medium-high sensitivity and specificity values (Fig. 3) and its AUC was 0.752, showing medium-high predictive efficiency. In Fig. 4, END predictive ability of serum MANF levels resembled those of NIHSS scores and hematoma volume (both *P* > 0.05). Intriguingly, serum MANF levels combined with NIHSS scores and hematoma volume showed insignificantly higher END predictive capability than NIHSS scores and hematoma volume alone (both *P* > 0.05).

**Fig. 5:**
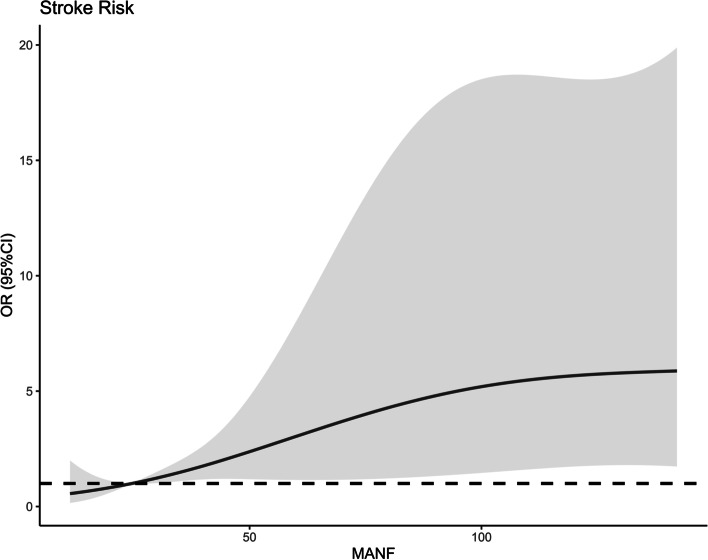
Restricted cubic spline for judging a linear relationship between serum mesencephalic astrocyte-derived neurotrophic factor levels and early neurologic deterioration after acute intracerebral hemorrhage. A linear relationship existed between serum mesencephalic astrocyte-derived neurotrophic factor levels and early neurologic deterioration after acute intracerebral hemorrhage (*P*>0.05). OR indicates odds ratio; 95% CI, 95% confidence interval; MANF, mesencephalic astrocyte-derived neurotrophic factor

**Fig. 6:**
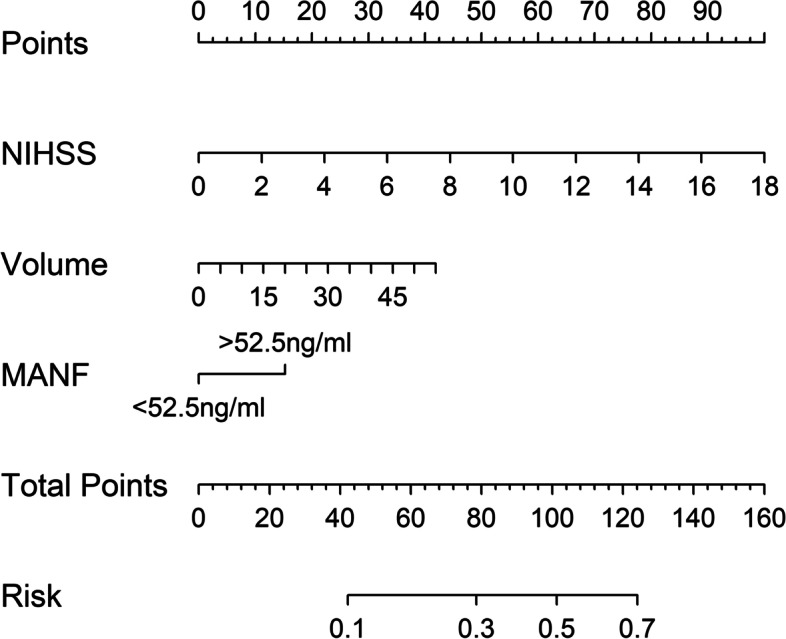
Nomogram of combination model for predicting early neurologic deterioration after acute intracerebral hemorrhage. A total of points were calculated to judge risk of early neurologic deterioration after acute intracerebral hemorrhage, based on serum mesencephalic astrocyte-derived neurotrophic factor levels, National Institutes of Health Stroke Scale scores and hematoma volume. MANF denotes mesencephalic astrocyte-derived neurotrophic factor; NIHSS, National Institutes of Health Stroke Scale; Volume, hematoma volume

**Fig. 7:**
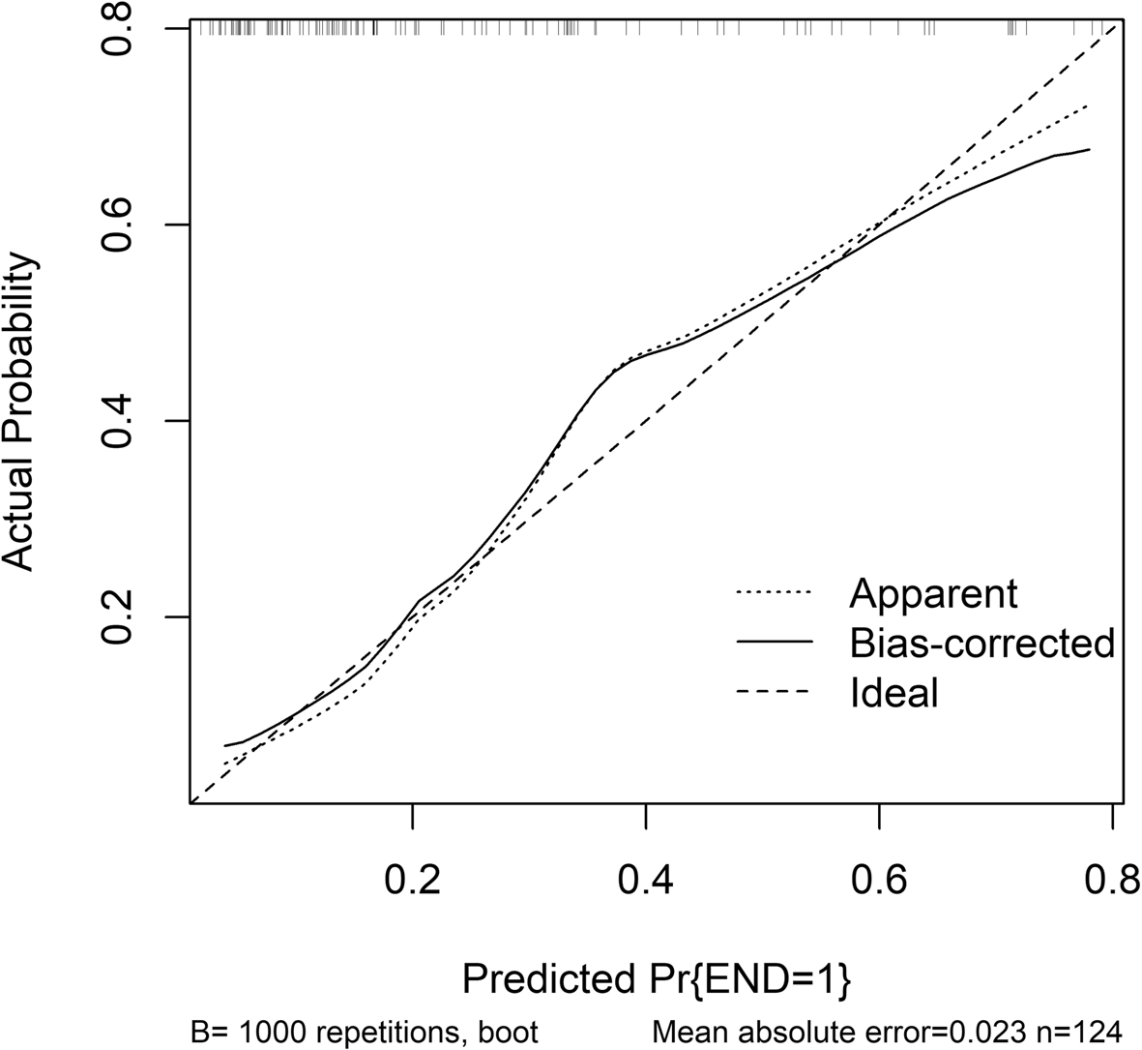
Calibration curve of combination model for predicting early neurologic deterioration after acute intracerebral hemorrhage. There was a relatively high stability of the combination model for predicting early neurologic deterioration after acute intracerebral hemorrhage based on serum mesencephalic astrocyte-derived neurotrophic factor levels, National Institutes of Health Stroke Scale scores and hematoma volume. END indicates early neurologic deterioration

Figure 5 shows that a linear correlation existed between serum MANF levels and END (*P* = 0.212). There was a significantly higher percentage of serum MANF levels > 52.5 ng/ml (the cutoff value under ROC curve) in END patients than in non-END patients (*P* < 0.001; Table 2). Other END-related variables, including NIHSS scores, hematoma volumes, blood leucocyte counts and blood glucose levels, were displayed in Table 2 (all *P* < 0.05). When the preceding significantly END-associated factors were incorporated into the binary logistic regression model, it was confirmed that NIHSS scores (OR, 1.232; 95% CI, 1.015–1.495; *P* = 0.012), hematoma volumes (OR, 1.080; 95% CI, 1.027–1.135; *P* = 0.024) and serum MANF levels > 52.5 ng/ml (OR, 2.713; 95% CI, 1.004–7.330; *P* = 0.042) independently predicted END. In Fig. 6, the nomogram, wherein NIHSS scores, hematoma volumes and serum MANF levels > 52.5 ng/ml were combined, showed END predictive scores. Figure 7 displays that such a combination model could be at rather stability of predictive ability (using Hosmer & Lemeshow test, *P* = 0.724).

**Table 2:** Factors in relation to early neurologic deterioration after acute intracerebral hemorrhage

Variables	END	Non-END	*P* values
Number	33	91	
Age (years)	59.3 ± 11.5	62.3 ± 12.4	0.224
Gender (male/female)	16/17	53/38	0.334
Body mass index (kg/m^2^)	26.1 ± 3.1	25.5 ± 3.5	0.407
Hypertension	19 (57.6%)	55 (60.4%)	0.774
Diabetes mellitus	11 (33.3%)	17 (18.7%)	0.085
Hyperlipidemia	9 (27.3%)	29 (31.9%)	0.624
Coronary heart disease	2 (6.1%)	7 (7.7%)	1.000
Cigarette smoking	16 (48.5%)	28(30.8%)	0.068
Alcohol drinking	14 (42.4%)	28 (30.8%)	0.226
Pretreatment of statins	9 (27.3%)	22 (24.2%)	0.725
Pretreatment of anticoagulation drugs	1 (3.3%)	4 (4.4%)	1.000
Pretreatment of antiplatelet drugs	6 (18.2%)	10 (11.0%)	0.363
Pretreatment of antihypertensive drugs	19 (57.6%)	48 (52.7%)	0.634
Pretreatment of hypoglycemic drugs or insulin	9 (27.3%)	12 (13.2%)	0.065
Admission time (h)	10.1 (7.6–15.0)	9.2 (5.8–12.6)	0.292
Blood-collection time (h)	11.8 (8.8–17.5)	10.9 (6.9–14.0)	0.341
Systolic arterial pressure (mmHg)	141.2 ± 21.2	148.3 ± 23.5	0.126
Diastolic arterial pressure (mmHg)	85.0 ± 8.9	88.7 ± 11.4	0.093
Hemorrhage locations (lobar / deep)	7/26	25/66	0.481
Extension of hematoma into intraventricular cavity	12 (36.4%)	20 (22.0%)	0.106
Extension of hematoma into subarachnoidal space	5 (15.2%)	6 (6.6%)	0.160
NIHSS scores	11 (10–16)	7 (4–9)	< 0.001
Hematoma volume (ml)	26 (16–32)	13 (10–19)	< 0.001
Blood leucocyte count (×10^9^/l)	9.6 (8.1–11.5)	7.8 (6.4–9.7)	0.026
Blood glucose levels (mmol/l)	13.8 (10.2–21.5)	10.7 (9.0-12.6)	0.002
Serum MANF levels > 52.5 ng/ml	21 (63.3%)	21 (23.1%)	< 0.001

Variables were shown as count (percentage), mean ± standard deviation or median (upper-lower quartile) where appropriate. Intergroup comparisons were done using the Chi-square test, Fisher exact test, Student’s 𝑡-test or Mann-Whitney test as appropriate. *NIHSS* indicates National Institutes of Health Stroke Scale, *MANF *Mesencephalic astrocyte-derived neurotrophic factor, *END *Early neurologic deterioration

### Serum MANF levels in independent association with post-stroke poor 90-day prognosis

**Fig. 8:**
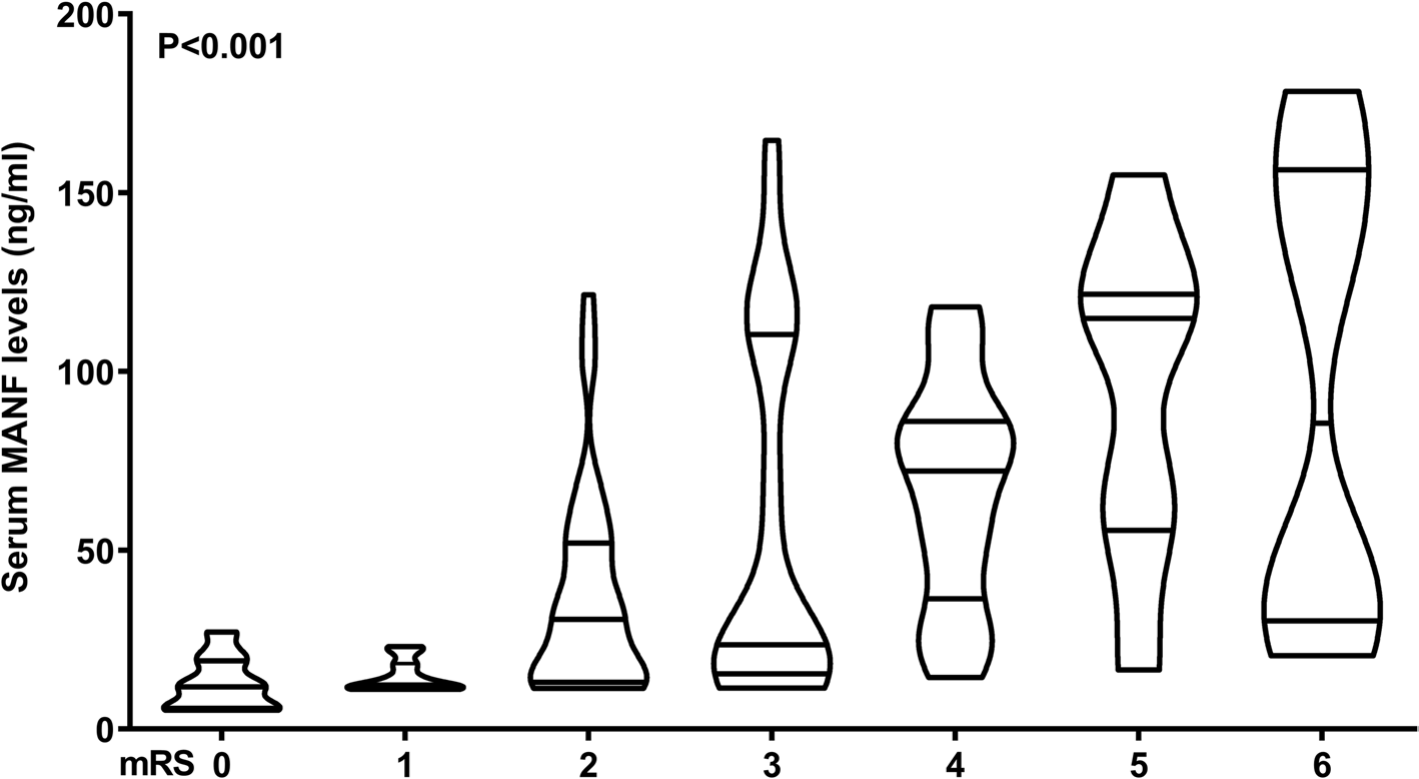
Relationship between serum mesencephalic astrocyte-derived neurotrophic factor levels and modified Rankin scale scores at 90 days after acute intracerebral hemorrhage. Serum mesencephalic astrocyte-derived neurotrophic factor levels were significantly lowest in patients with modified Rankin scale score 0, followed by scores from 1 to 5, and were substantially highest in those with score 6 at 90 days following acute intracerebral hemorrhage (*P*<0.001). MANF denotes mesencephalic astrocyte-derived neurotrophic factor; mRS, modified Rankin scale

At 90 days after ICH, mRS scores ranged from 0 to 6, with a median value of 2 (percentiles 25th – 75th, 2–4). Just as exhibited in Table 3, mRS scores were highly correlated with serum MANF levels (*P* < 0.001), NIHSS scores (*P* < 0.001), hematoma volumes (*P* < 0.001), extension of hematoma into intraventricular cavity (*P* < 0.05), blood leucocyte levels (*P* < 0.05) and blood glucose levels (*P* < 0.01). The above-mentioned six factors were forced into the multivariate linear regression model, and subsequently, 90-day mRS scores were independently correlated with serum MANF levels (beta, 0.018; 95% CI, 0.013–0.023; VIF = 1.984; t = 2.047; *P* = 0.043), NIHSS scores (beta, 0.138; 95% CI, 0.057–0.219; VIF = 2.948; t = 3.377; *P* = 0.001) and hematoma volumes (beta, 0.038; 95% CI, 0.004–0.072; VIF = 2.029; t = 2.228; *P* = 0.028). In addition, among this group of patients of ICH, 10 patients had mRS score 0; 16, mRS score 1; 37, mRS score 2; 28, mRS score 3; 16, mRS score 4; 7, mRS score 5; and 10, mRS score 6. In Fig. 8, serum MANF levels were markedly increased in order of mRS scores from 0 to 6 (*P* < 0.001).

**Table 3:** Factors in correlation with 90-day modified Rankin scale scores after acute intracerebral hemorrhage

Variables	*ρ*	*P* value
Age (years)	-0.009	0.921
Gender (male/female)	-0.105	0.245
Body mass index (kg/m^2^)	0.047	0.601
Hypertension	-0.032	0.721
Diabetes mellitus	0.116	0.201
Hyperlipidemia	0.037	0.681
Coronary heart disease	0.048	0.600
Cigarette smoking	0.083	0.360
Alcohol drinking	0.142	0.116
Pretreatment of statins	0.089	0.328
Pretreatment of anticoagulation drugs	-0.019	0.831
Pretreatment of antiplatelet drugs	-0.028	0.756
Pretreatment of antihypertensive drugs	0.102	0.258
Pretreatment of hypoglycemic drugs or insulin	-0.069	0.448
Admission time (h)	0.132	0.145
Blood-collection time (h)	0.143	0.113
Systolic arterial pressure (mmHg)	-0.064	0.482
Diastolic arterial pressure (mmHg)	-0.016	0.860
Hemorrhage locations (lobar / deep)	-0.009	0.919
Extension of hematoma into intraventricular cavity	0.185	0.039
Extension of hematoma into subarachnoidal space	0.123	0.174
NIHSS scores	0.691	< 0.001
Hematoma volume (ml)	0.688	< 0.001
Blood leucocyte count (×10^9^/l)	0.227	0.011
Blood glucose levels (mmol/l)	0.292	0.001
Serum MANF levels (ng/ml)	0.613	< 0.001

Using Spearman’s correlation coefficient test, correlations were reported as ρ values. *NIHSS* indicates National Institutes of Health Stroke Scale;

In aggregate, 61 ICH patients experienced a poor 90-day prognosis after stroke. Serum MANF levels ranged from 11.5 to 178.4 ng/ml (median, 62.1 ng/ml; upper-lower quartiles, 22.9-114.8 ng/ml) in patients experiencing a poor prognosis and from 5.3 to 121.4 ng/ml (median, 16.4 ng/ml; upper-lower quartiles, 11.8–33.1 ng/ml) in those with a good prognosis. Obviously, serum MANF levels were profoundly elevated in poor prognosis patients, as compared to good prognosis ones (*P* < 0.001).

**Fig. 9:**
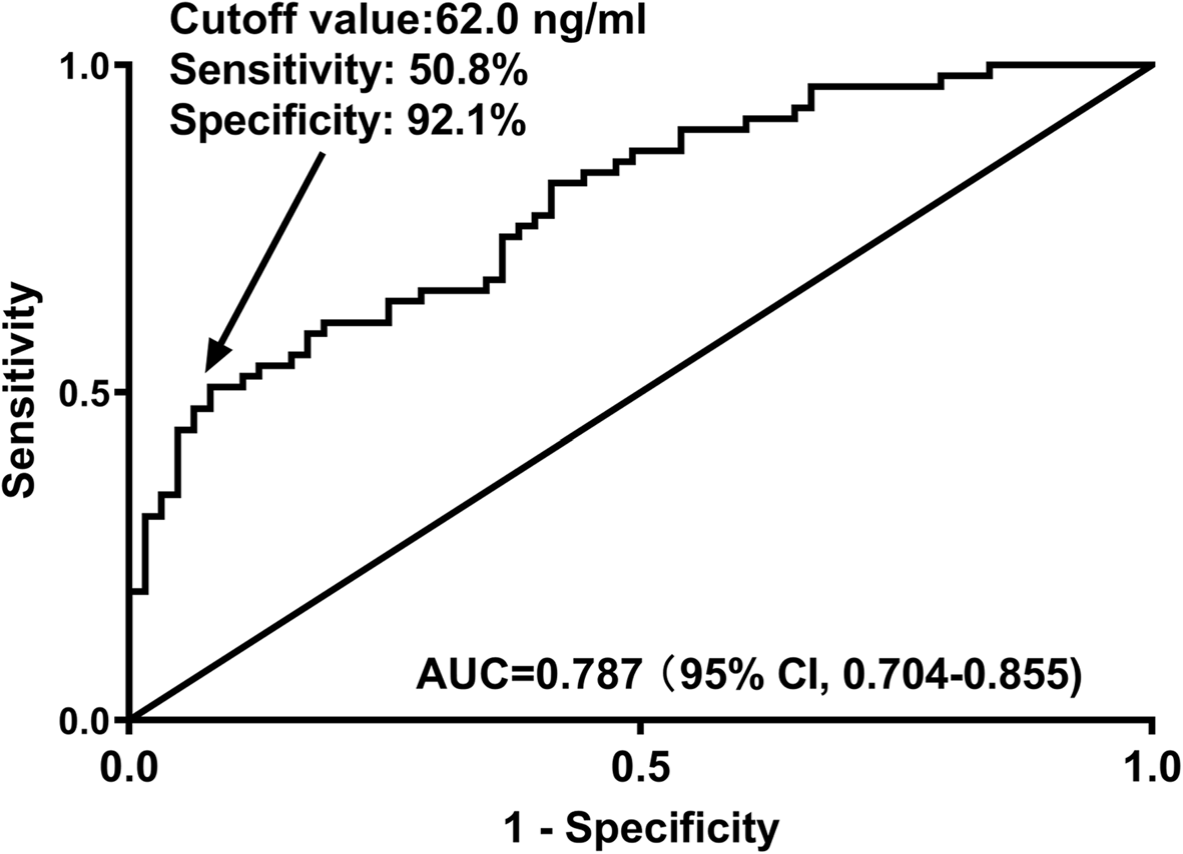
Receiver operating characteristic curve of serum mesencephalic astrocyte-derived neurotrophic factor levels for predicting poor 90-day prognosis after acute supratentorial intracerebral hemorrhage. Serum mesencephalic astrocyte-derived neurotrophic factor levels significantly distinguished patients at risk of poor 90-day prognosis following acute intracerebral hemorrhage and using Youden method, an optimal value was selected, which predicted such an adverse affair with medium-high sensitivity and specificity values (*P*<0.001). AUC means area under curve; 95% CI, 95% confidence interval

**Fig. 10:**
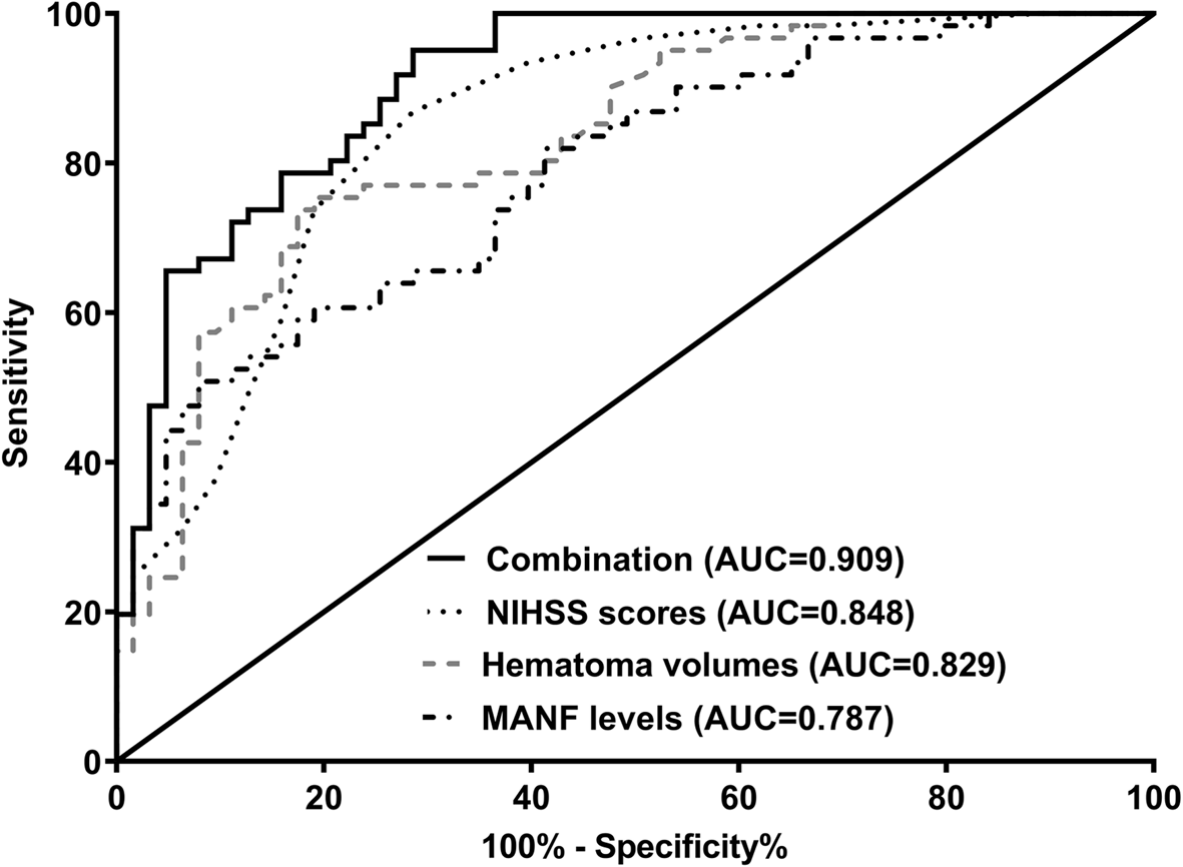
Predictive ability with respect to combination model for poor 90-day prognosis after acute supratentorial intracerebral hemorrhage. Predictive ability of serum mesencephalic astrocyte-derived neurotrophic factor levels for poor 90-day prognosis was similar to those of National Institutes of Health Stroke Scale scores and hematoma volume (both *P*>0.05). Serum mesencephalic astrocyte-derived neurotrophic factor levels combined with National Institutes of Health Stroke Scale scores and hematoma volume showed significantly higher predictive capability than National Institutes of Health Stroke Scale scores and hematoma volume alone (both*P*>0.05). MANF denotes mesencephalic astrocyte-derived neurotrophic factor; NIHSS, National Institutes of Health Stroke Scale; AUC, area under curve

Under ROC curve, serum MANF levels significantly discriminated patients with development of poor prognosis (*P* < 0.001; Fig. 9). Using Youden method, the optimal cutoff value of serum MANF levels was identified, and its levels > 62.0 ng/ml distinguished patients at risk of poor prognosis with medium-high sensitivity and specificity values (Fig. 9). In Fig. 10, prognostic predictive capability of serum MANF levels was equivalent to those of NIHSS scores and hematoma volume (both *P* > 0.05). Interestingly, prognostic predictive ability of combination of serum MANF levels with NIHSS scores and hematoma volume was substantially superior to that of NIHSS scores and hematoma volume alone (both *P* < 0.05).

**Fig. 11:**
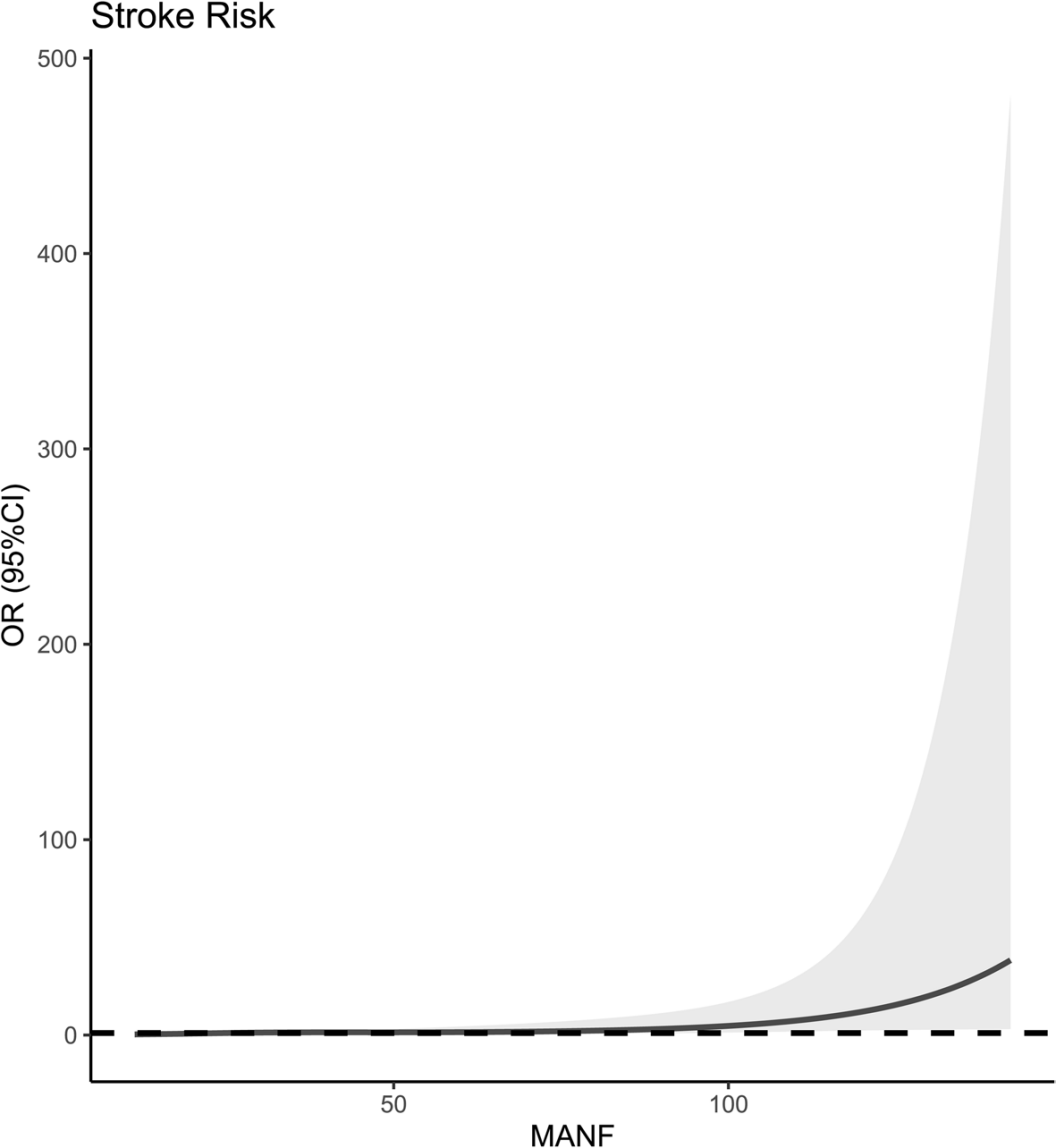
Restricted cubic spline for judging a linear relationship between serum mesencephalic astrocyte-derived neurotrophic factor levels and poor 90-day prognosis after acute intracerebral hemorrhage. There was a linear relationship between serum mesencephalic astrocyte-derived neurotrophic factor levels and poor 90-day prognosis after acute intracerebral hemorrhage (*P*>0.05). OR indicates odds ratio; 95% CI, 95% confidence interval; MANF, mesencephalic astrocyte-derived neurotrophic factor

**Fig. 12:**
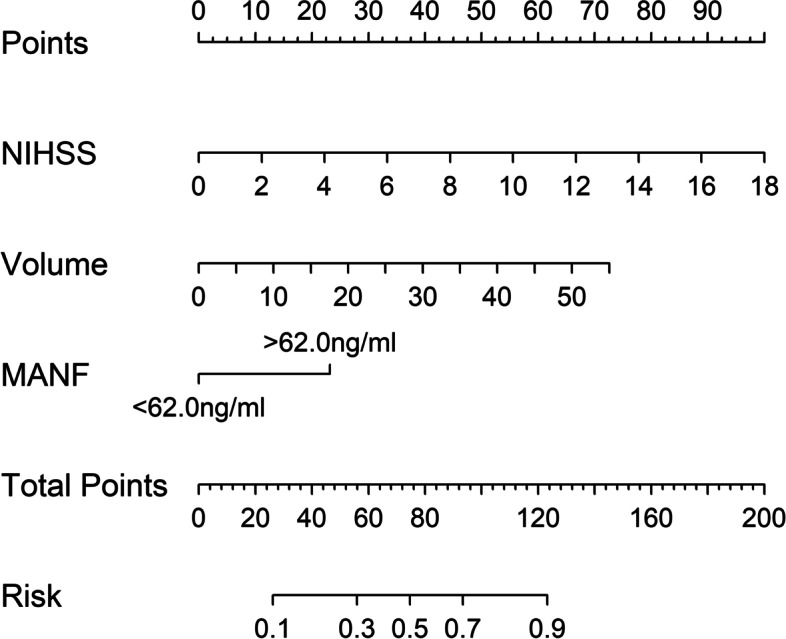
Nomogram of combination model for predicting poor 90-day prognosis after acute intracerebral hemorrhage. A total of points were calculated to judge risk of poor 90-day prognosis after acute intracerebral hemorrhage, based on serum mesencephalic astrocyte-derived neurotrophic factor levels, National Institutes of Health Stroke Scale scores and hematoma volume. MANF denotes mesencephalic astrocyte-derived neurotrophic factor; NIHSS, National Institutes of Health Stroke Scale; Volume, hematoma volume

**Fig. 13:**
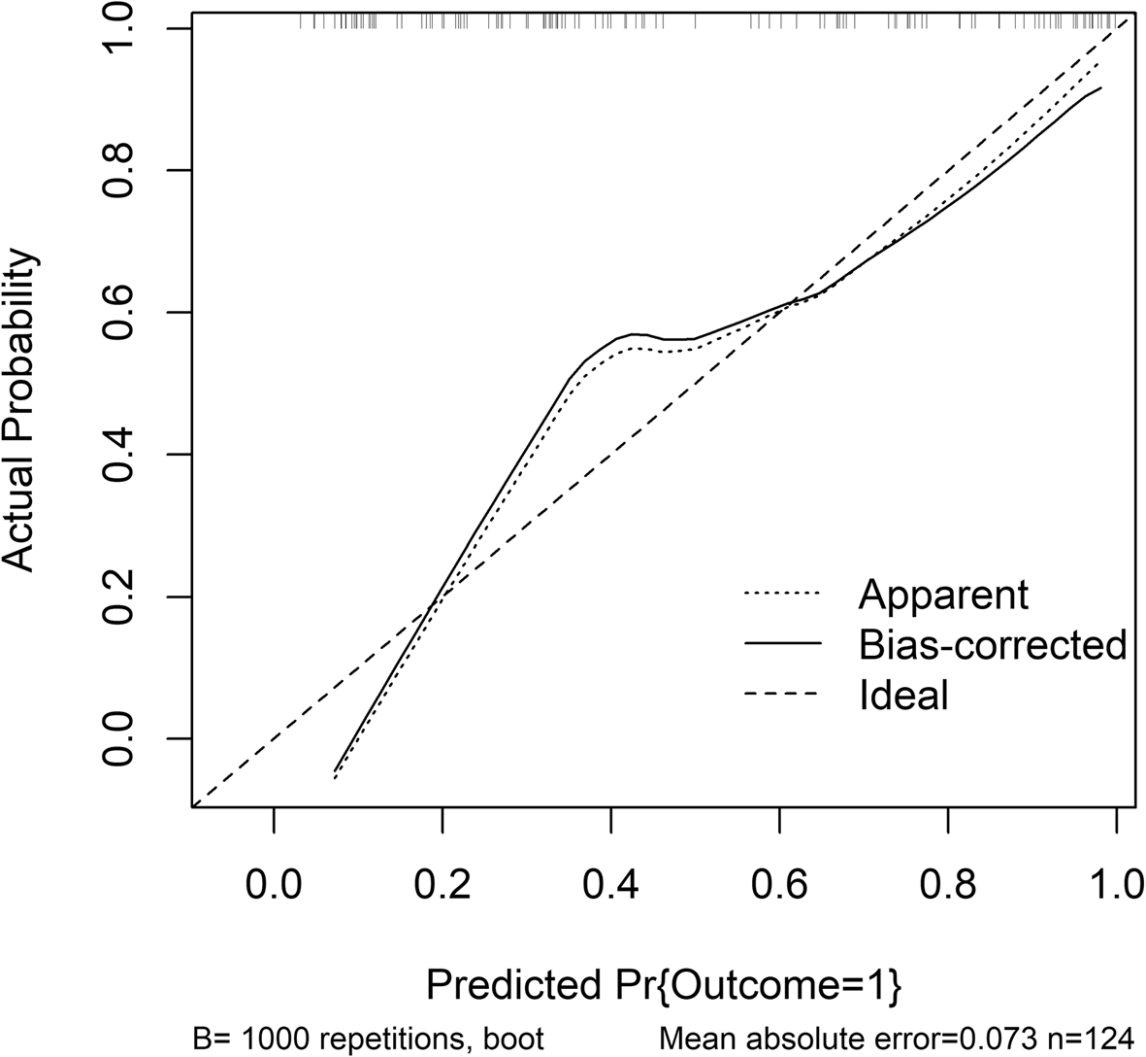
Calibration curve of combination model for predicting poor 90-day prognosis after acute intracerebral hemorrhage. There was a relatively high stability of the combination model for predicting poor 90-day prognosis after acute intracerebral hemorrhage based on serum mesencephalic astrocyte-derived neurotrophic factor levels, National Institutes of Health Stroke Scale scores and hematoma volume

Using restricted cubic spline, there was a linear correlation between serum MANF levels and poor prognosis (*P* = 0.296; Fig. 11). Based on the cutoff value of serum MANF levels (62.0 ng/ml), patients were dichotomized. In Table 4, factors, which were dramatically associated with a poor prognosis, were serum MANF levels > 62.0 ng/ml, NIHSS scores, hematoma volumes and blood glucose levels (all *P* < 0.05). Using multivariate analysis, NIHSS scores (OR, 1.286; 95% CI, 1.065–1.552; *P* = 0.009), hematoma volumes (OR, 1.136; 95% CI, 1.068–1.209; *P* = 0.014) and serum MANF levels > 62.0 ng/ml (OR, 3.848; 95% CI, 1.193–12.417; *P* = 0.024) were independently associated with a poor prognosis after stroke. In Fig. 12, the nomogram, which showed combination of NIHSS scores, hematoma volumes and serum MANF levels > 62.0 ng/ml, was established to predict a poor 90-day prognosis. In Fig. 13, such a combination model was verified to be stable significantly (using Hosmer & Lemeshow test, *P* = 0.869).

**Table 4:** Factors in relation to 90-day poor prognosis after acute intracerebral hemorrhage

Variables	Poor prognosis	Good prognosis	*P* value
Number	61	63	
Age (years)	61.7 ± 11.8	61.3 ± 12.7	0.826
Gender (male/female)	29/32	40/23	0.074
Body mass index (kg/m^2^)	25.8 ± 3.4	25.6 ± 3.5	0.828
Hypertension	37 (60.7%)	37 (58.7%)	0.827
Diabetes mellitus	12 (19.7%)	16 (25.4%)	0.446
Hyperlipidemia	19 (31.1%)	19 (30.2%)	0.905
Coronary heart disease	6 (9.8%)	3 (4.8%)	0.319
Cigarette smoking	22 (36.1%)	22 (34.9%)	0.894
Alcohol drinking	23 (37.7%)	19 (30.2%)	0.375
Pretreatment of statins	17 (27.9%)	14 (22.2%)	0.468
Pretreatment of anticoagulation drugs	3 (4.9%)	2 (3.2%)	0.677
Pretreatment of antiplatelet drugs	7 (11.5%)	9 (14.3%)	0.641
Pretreatment of antihypertensive drugs	35 (57.4%)	32(50.8%)	0.462
Pretreatment of hypoglycemic drugs or insulin	11 (18.0%)	10 (15.9%)	0.749
Admission time (h)	9.4 (7.0-12.6)	9.1 (5.3–14.9)	0.910
Blood-collection time (h)	11.1 (8.5–14.8)	10.8 (6.7–16.6)	0.787
Systolic arterial pressure (mmHg)	146.5 ± 23.6	146.3 ± 22.7	0.967
Diastolic arterial pressure (mmHg)	88.0 ± 11.1	87.5 ± 10.8	0.784
Hemorrhage locations (lobar / deep)	17/44	15/48	0.606
Extension of hematoma into intraventricular cavity	19 (31.1%)	13 (20.6%)	0.181
Extension of hematoma into subarachnoidal space	8 (13.1%)	3 (4.8%)	0.102
NIHSS scores	10 (8–13)	6 (3–8)	< 0.001
Hematoma volume (ml)	23 (16–29)	11 (7–15)	< 0.001
Blood leucocyte count (×10^9^/l)	8.8 (6.8–11.3)	7.6 (6.3–9.5)	0.057
Blood glucose levels (mmol/l)	12.8 (9.6–17.2)	10.7 (9.2–12.4)	0.017
Serum MANF levels > 62.0 ng/ml	30 (49.2%)	5 (7.9%)	< 0.001

Variables were shown as count (percentage), mean ± standard deviation or median (upper-lower quartile) where appropriate. Intergroup comparisons were done using the Chi-square test, Fisher exact test, Student’s 𝑡-test or Mann-Whitney test as appropriate. *NIHSS* indicates National Institutes of Health Stroke Scale, *MANF *Mesencephalic astrocyte-derived neurotrophic factor. Poor prognosis was referred to as modified Rankin Scale scores of 3–6

## Discussion

To the best of our knowledge, there is a paucity of data available regarding circulating MANF levels after ICH and its prognostic role in ICH. The main findings of our study were that: (1) raised serum MANF levels after ICH were independently correlated with stroke severity indicated by NIHSS scores and hematoma volumes, as well as were independently associated with clinical outcome reflected by END, mRS scores and poor prognosis at 90 days after stroke; (2) Serum MANF levels predicted END and poor 90-day prognosis at AUCs of 0.752 and 0.787 respectively, and displayed similar predictive ability, as compared to NIHSS scores and hematoma volumes; (3) combination of serum MANF levels with NIHSS scores and hematoma volumes showed a comparatively high prognostic ability and a relatively stable prognostic efficiency based on ROC curve, nomogram, calibration curve and restricted cubic spline. In summary, serum Nrf2 may be of clinical value to be used as a prognostic biomarker in acute ICH.

ICH is a complicated cerebrovascular disease, wherein endoplasmic reticulum stress contributes to the neuroinflammation, thereby resulting in brain edema, neuronal injury and finally neurologic impairment [19]. MANF, which is extensively expressed in both neurons and non-neuronal tissues, is dominantly localized in the endoplasmic reticulum lumen and is quickly secreted from cells upon endoplasmic reticulum stress [20]. Experimental data showed that MANF obviously alleviated acute traumatic, ischemic and hemorrhagic brain injury via inhibiting neuroinflammation, depressing neuronal apoptosis and ameliorating blood-brain barrier disruption, indicating that endogenous MANF expression may harbor neuroprotective property [11–14].

There was an obvious enhancement of serum levels of MANF in both humans and rats with ischemic stroke [15]. Consistently, serum MANF levels were substantially elevated in this group of ICH patients. Expressions of MANF mRNA in blood cells insignificantly differed between controls and patients with acute ischemic stroke [15]. Similarly, no significant differences were found of mRNA expressions in blood cells between controls and ischemic stroke rats [15]. It is deduced that serum MANF may not originate from blood cells. However, MANF was obviously expressed in brain tissues and its expressions were markedly up-regulated after acute brain injury [11–14]. Thus, serum MANF may be at least partially derived from damaged brain tissues. Nevertheless, MANF is broadly expressed in endocrine islet beta-cells, cardiac myocytes, circulating immune cells and neuro-endocrine system [21, 22]. Conceivably, serum MANF may partially originate from other peripheral tissues or cells.

A recent study of 50 acute ischemic stroke patients, 56 transient ischemic attack patients and 48 controls have demonstrated that elevated serum MANF levels were highly correlated with NIHSS scores and Alberta Stroke Program Early CT Scores, as well as were highly associated with occurrence of transient ischemic attack [15]. In our study including 124 ICH patients and the same number of controls, serum MANF levels, in close correlation with severity, were highly associated with END and poor prognosis after ICH. It is noteworthy that all correlations or associations of serum MANF levels with severity and prognosis were confirmed using multivariate analyses. Moreover, restricted cubic spline was utilized to confirm linear correlation of serum MANF levels with END and prognosis. In other words, logistic regression model is optimal for multivariate analysis. Alternatively, two combination models shown as nomograms, which contained serum MANF levels, NIHSS scores and hematoma volumes, were built to discriminate risk of END and a poor prognosis. Predictive ability and stability of such models were verified under ROC curve and calibration curve. Overall, serum MANF may be worthy of being considered as a promising biochemical marker of ICH.

There are several weaknesses and strengths in this study. To the best of our knowledge, this is a first series for investigating circulating MANF levels in patients with ICH and thereafter finding its close correlation with severity and its significant association with clinical outcome of ICH. And, all correlations and associations between serum MANF levels and severity plus prognosis were verified using multivariate analyses. However, our study recruited 124 patients with ICH, meaning a medium sample size, and therefore the conclusions should be validated by a larger cohort study.

## Conclusions

Since MANF is in possession of neuroprotective effects, this clinical epidemiological investigation was implemented to determine whether serum MANF levels are altered after human ICH. It is found that serum MANF levels are significantly increased, have independent correlation with admission NIHSS scores, baseline hematoma volumes and post-stroke 90-day mRS scores, are associated with END and poor 90-day prognosis independently, show efficient prognostic predictive potential and exhibit additive effect of prognostic ability when combined with NIHSS scores and hematoma scores. Conceivably, such data are strongly supportive of the notion that serum MANF may be accurately reflective of stroke severity and neurological outcome of human ICH, as well as its determination may be of clinical significance.

## Data Availability

The datasets generated and/or analyzed during the current study are not publicly available due for they are personal data but are available from the corresponding author on reasonable request.
